# Centrality dependence of charged jet production in p–Pb collisions at $\sqrt{s_\mathrm{NN}}$ = 5.02 TeV

**DOI:** 10.1140/epjc/s10052-016-4107-8

**Published:** 2016-05-17

**Authors:** Alice Collaboration, J. Adam, D. Adamová, M. M. Aggarwal, G. Aglieri Rinella, M. Agnello, N. Agrawal, Z. Ahammed, S. Ahmad, S. U. Ahn, S. Aiola, A. Akindinov, S. N. Alam, D. S. D. Albuquerque, D. Aleksandrov, B. Alessandro, D. Alexandre, R. Alfaro Molina, A. Alici, A. Alkin, J. R. M. Almaraz, J. Alme, T. Alt, S. Altinpinar, I. Altsybeev, C. Alves Garcia Prado, C. Andrei, A. Andronic, V. Anguelov, T. Antičić, F. Antinori, P. Antonioli, L. Aphecetche, H. Appelshäuser, S. Arcelli, R. Arnaldi, O. W. Arnold, I. C. Arsene, M. Arslandok, B. Audurier, A. Augustinus, R. Averbeck, M. D. Azmi, A. Badalà, Y. W. Baek, S. Bagnasco, R. Bailhache, R. Bala, S. Balasubramanian, A. Baldisseri, R. C. Baral, A. M. Barbano, R. Barbera, F. Barile, G. G. Barnaföldi, L. S. Barnby, V. Barret, P. Bartalini, K. Barth, J. Bartke, E. Bartsch, M. Basile, N. Bastid, S. Basu, B. Bathen, G. Batigne, A. Batista Camejo, B. Batyunya, P. C. Batzing, I. G. Bearden, H. Beck, C. Bedda, N. K. Behera, I. Belikov, F. Bellini, H. Bello Martinez, R. Bellwied, R. Belmont, E. Belmont-Moreno, V. Belyaev, G. Bencedi, S. Beole, I. Berceanu, A. Bercuci, Y. Berdnikov, D. Berenyi, R. A. Bertens, D. Berzano, L. Betev, A. Bhasin, I. R. Bhat, A. K. Bhati, B. Bhattacharjee, J. Bhom, L. Bianchi, N. Bianchi, C. Bianchin, J. Bielčík, J. Bielčíková, A. Bilandzic, G. Biro, R. Biswas, S. Biswas, S. Bjelogrlic, J. T. Blair, D. Blau, C. Blume, F. Bock, A. Bogdanov, H. Bøggild, L. Boldizsár, M. Bombara, J. Book, H. Borel, A. Borissov, M. Borri, F. Bossú, E. Botta, C. Bourjau, P. Braun-Munzinger, M. Bregant, T. Breitner, T. A. Broker, T. A. Browning, M. Broz, E. J. Brucken, E. Bruna, G. E. Bruno, D. Budnikov, H. Buesching, S. Bufalino, P. Buncic, O. Busch, Z. Buthelezi, J. B. Butt, J. T. Buxton, J. Cabala, D. Caffarri, X. Cai, H. Caines, L. Calero Diaz, A. Caliva, E. Calvo Villar, P. Camerini, F. Carena, W. Carena, F. Carnesecchi, J. Castillo Castellanos, A. J. Castro, E. A. R. Casula, C. Ceballos Sanchez, J. Cepila, P. Cerello, J. Cerkala, B. Chang, S. Chapeland, M. Chartier, J. L. Charvet, S. Chattopadhyay, S. Chattopadhyay, A. Chauvin, V. Chelnokov, M. Cherney, C. Cheshkov, B. Cheynis, V. Chibante Barroso, D. D. Chinellato, S. Cho, P. Chochula, K. Choi, M. Chojnacki, S. Choudhury, P. Christakoglou, C. H. Christensen, P. Christiansen, T. Chujo, S. U. Chung, C. Cicalo, L. Cifarelli, F. Cindolo, J. Cleymans, F. Colamaria, D. Colella, A. Collu, M. Colocci, G. Conesa Balbastre, Z. Conesa del Valle, M. E. Connors, J. G. Contreras, T. M. Cormier, Y. Corrales Morales, I. Cortés Maldonado, P. Cortese, M. R. Cosentino, F. Costa, P. Crochet, R. Cruz Albino, E. Cuautle, L. Cunqueiro, T. Dahms, A. Dainese, M. C. Danisch, A. Danu, D. Das, I. Das, S. Das, A. Dash, S. Dash, S. De, A. De Caro, G. de Cataldo, C. de Conti, J. de Cuveland, A. De Falco, D. De Gruttola, N. De Marco, S. De Pasquale, A. Deisting, A. Deloff, E. Dénes, C. Deplano, P. Dhankher, D. Di Bari, A. Di Mauro, P. Di Nezza, M. A. Diaz Corchero, T. Dietel, P. Dillenseger, R. Divià, Ø. Djuvsland, A. Dobrin, D. Domenicis Gimenez, B. Dönigus, O. Dordic, T. Drozhzhova, A. K. Dubey, A. Dubla, L. Ducroux, P. Dupieux, R. J. Ehlers, D. Elia, E. Endress, H. Engel, E. Epple, B. Erazmus, I. Erdemir, F. Erhardt, B. Espagnon, M. Estienne, S. Esumi, J. Eum, D. Evans, S. Evdokimov, G. Eyyubova, L. Fabbietti, D. Fabris, J. Faivre, A. Fantoni, M. Fasel, L. Feldkamp, A. Feliciello, G. Feofilov, J. Ferencei, A. Fernández Téllez, E. G. Ferreiro, A. Ferretti, A. Festanti, V. J. G. Feuillard, J. Figiel, M. A. S. Figueredo, S. Filchagin, D. Finogeev, F. M. Fionda, E. M. Fiore, M. G. Fleck, M. Floris, S. Foertsch, P. Foka, S. Fokin, E. Fragiacomo, A. Francescon, U. Frankenfeld, G. G. Fronze, U. Fuchs, C. Furget, A. Furs, M. Fusco Girard, J. J. Gaardhøje, M. Gagliardi, A. M. Gago, M. Gallio, D. R. Gangadharan, P. Ganoti, C. Gao, C. Garabatos, E. Garcia-Solis, C. Gargiulo, P. Gasik, E. F. Gauger, M. Germain, M. Gheata, P. Ghosh, S. K. Ghosh, P. Gianotti, P. Giubellino, P. Giubilato, E. Gladysz-Dziadus, P. Glässel, D. M. Goméz Coral, A. Gomez Ramirez, A. S. Gonzalez, V. Gonzalez, P. González-Zamora, S. Gorbunov, L. Görlich, S. Gotovac, V. Grabski, O. A. Grachov, L. K. Graczykowski, K. L. Graham, A. Grelli, A. Grigoras, C. Grigoras, V. Grigoriev, A. Grigoryan, S. Grigoryan, B. Grinyov, N. Grion, J. M. Gronefeld, J. F. Grosse-Oetringhaus, R. Grosso, F. Guber, R. Guernane, B. Guerzoni, K. Gulbrandsen, T. Gunji, A. Gupta, R. Gupta, R. Haake, Ø. Haaland, C. Hadjidakis, M. Haiduc, H. Hamagaki, G. Hamar, J. C. Hamon, J. W. Harris, A. Harton, D. Hatzifotiadou, S. Hayashi, S. T. Heckel, E. Hellbär, H. Helstrup, A. Herghelegiu, G. Herrera Corral, B. A. Hess, K. F. Hetland, H. Hillemanns, B. Hippolyte, D. Horak, R. Hosokawa, P. Hristov, T. J. Humanic, N. Hussain, T. Hussain, D. Hutter, D. S. Hwang, R. Ilkaev, M. Inaba, E. Incani, M. Ippolitov, M. Irfan, M. Ivanov, V. Ivanov, V. Izucheev, N. Jacazio, P. M. Jacobs, M. B. Jadhav, S. Jadlovska, J. Jadlovsky, C. Jahnke, M. J. Jakubowska, H. J. Jang, M. A. Janik, P. H. S. Y. Jayarathna, C. Jena, S. Jena, R. T. Jimenez Bustamante, P. G. Jones, A. Jusko, P. Kalinak, A. Kalweit, J. Kamin, J. H. Kang, V. Kaplin, S. Kar, A. Karasu Uysal, O. Karavichev, T. Karavicheva, L. Karayan, E. Karpechev, U. Kebschull, R. Keidel, D. L. D. Keijdener, M. Keil, M. Mohisin Khan, P. Khan, S. A. Khan, A. Khanzadeev, Y. Kharlov, B. Kileng, D. W. Kim, D. J. Kim, D. Kim, H. Kim, J. S. Kim, M. Kim, S. Kim, T. Kim, S. Kirsch, I. Kisel, S. Kiselev, A. Kisiel, G. Kiss, J. L. Klay, C. Klein, J. Klein, C. Klein-Bösing, S. Klewin, A. Kluge, M. L. Knichel, A. G. Knospe, C. Kobdaj, M. Kofarago, T. Kollegger, A. Kolojvari, V. Kondratiev, N. Kondratyeva, E. Kondratyuk, A. Konevskikh, M. Kopcik, P. Kostarakis, M. Kour, C. Kouzinopoulos, O. Kovalenko, V. Kovalenko, M. Kowalski, G. Koyithatta Meethaleveedu, I. Králik, A. Kravčáková, M. Krivda, F. Krizek, E. Kryshen, M. Krzewicki, A. M. Kubera, V. Kučera, C. Kuhn, P. G. Kuijer, A. Kumar, J. Kumar, L. Kumar, S. Kumar, P. Kurashvili, A. Kurepin, A. B. Kurepin, A. Kuryakin, M. J. Kweon, Y. Kwon, S. L. La Pointe, P. La Rocca, P. Ladron de Guevara, C. Lagana Fernandes, I. Lakomov, R. Langoy, K. Lapidus, C. Lara, A. Lardeux, A. Lattuca, E. Laudi, R. Lea, L. Leardini, G. R. Lee, S. Lee, F. Lehas, S. Lehner, R. C. Lemmon, V. Lenti, E. Leogrande, I. León Monzón, H. León Vargas, M. Leoncino, P. Lévai, S. Li, X. Li, J. Lien, R. Lietava, S. Lindal, V. Lindenstruth, C. Lippmann, M. A. Lisa, H. M. Ljunggren, D. F. Lodato, P. I. Loenne, V. Loginov, C. Loizides, X. Lopez, E. López Torres, A. Lowe, P. Luettig, M. Lunardon, G. Luparello, T. H. Lutz, A. Maevskaya, M. Mager, S. Mahajan, S. M. Mahmood, A. Maire, R. D. Majka, M. Malaev, I. Maldonado Cervantes, L. Malinina, D. Mal’Kevich, P. Malzacher, A. Mamonov, V. Manko, F. Manso, V. Manzari, M. Marchisone, J. Mareš, G. V. Margagliotti, A. Margotti, J. Margutti, A. Marín, C. Markert, M. Marquard, N. A. Martin, J. Martin Blanco, P. Martinengo, M. I. Martínez, G. Martínez García, M. Martinez Pedreira, A. Mas, S. Masciocchi, M. Masera, A. Masoni, A. Mastroserio, A. Matyja, C. Mayer, J. Mazer, M. A. Mazzoni, D. Mcdonald, F. Meddi, Y. Melikyan, A. Menchaca-Rocha, E. Meninno, J. Mercado Pérez, M. Meres, Y. Miake, M. M. Mieskolainen, K. Mikhaylov, L. Milano, J. Milosevic, A. Mischke, A. N. Mishra, D. Miśkowiec, J. Mitra, C. M. Mitu, N. Mohammadi, B. Mohanty, L. Molnar, L. Montaño Zetina, E. Montes, D. A. Moreira De Godoy, L. A. P. Moreno, S. Moretto, A. Morreale, A. Morsch, V. Muccifora, E. Mudnic, D. Mühlheim, S. Muhuri, M. Mukherjee, J. D. Mulligan, M. G. Munhoz, R. H. Munzer, H. Murakami, S. Murray, L. Musa, J. Musinsky, B. Naik, R. Nair, B. K. Nandi, R. Nania, E. Nappi, M. U. Naru, H. Natal da Luz, C. Nattrass, S. R. Navarro, K. Nayak, R. Nayak, T. K. Nayak, S. Nazarenko, A. Nedosekin, L. Nellen, F. Ng, M. Nicassio, M. Niculescu, J. Niedziela, B. S. Nielsen, S. Nikolaev, S. Nikulin, V. Nikulin, F. Noferini, P. Nomokonov, G. Nooren, J. C. C. Noris, J. Norman, A. Nyanin, J. Nystrand, H. Oeschler, S. Oh, S. K. Oh, A. Ohlson, A. Okatan, T. Okubo, L. Olah, J. Oleniacz, A. C. Oliveira Da Silva, M. H. Oliver, J. Onderwaater, C. Oppedisano, R. Orava, M. Oravec, A. Ortiz Velasquez, A. Oskarsson, J. Otwinowski, K. Oyama, M. Ozdemir, Y. Pachmayer, D. Pagano, P. Pagano, G. Paić, S. K. Pal, J. Pan, A. K. Pandey, V. Papikyan, G. S. Pappalardo, P. Pareek, W. J. Park, S. Parmar, A. Passfeld, V. Paticchio, R. N. Patra, B. Paul, H. Pei, T. Peitzmann, H. Pereira Da Costa, D. Peresunko, E. Perez Lezama, V. Peskov, Y. Pestov, V. Petráček, V. Petrov, M. Petrovici, C. Petta, S. Piano, M. Pikna, P. Pillot, L. O. D. L. Pimentel, O. Pinazza, L. Pinsky, D. B. Piyarathna, M. Płoskoń, M. Planinic, J. Pluta, S. Pochybova, P. L. M. Podesta-Lerma, M. G. Poghosyan, B. Polichtchouk, N. Poljak, W. Poonsawat, A. Pop, S. Porteboeuf-Houssais, J. Porter, J. Pospisil, S. K. Prasad, R. Preghenella, F. Prino, C. A. Pruneau, I. Pshenichnov, M. Puccio, G. Puddu, P. Pujahari, V. Punin, J. Putschke, H. Qvigstad, A. Rachevski, S. Raha, S. Rajput, J. Rak, A. Rakotozafindrabe, L. Ramello, F. Rami, R. Raniwala, S. Raniwala, S. S. Räsänen, B. T. Rascanu, D. Rathee, K. F. Read, K. Redlich, R. J. Reed, A. Rehman, P. Reichelt, F. Reidt, X. Ren, R. Renfordt, A. R. Reolon, A. Reshetin, K. Reygers, V. Riabov, R. A. Ricci, T. Richert, M. Richter, P. Riedler, W. Riegler, F. Riggi, C. Ristea, E. Rocco, M. Rodríguez Cahuantzi, A. Rodriguez Manso, K. Røed, E. Rogochaya, D. Rohr, D. Röhrich, F. Ronchetti, L. Ronflette, P. Rosnet, A. Rossi, F. Roukoutakis, A. Roy, C. Roy, P. Roy, A. J. Rubio Montero, R. Rui, R. Russo, B. D. Ruzza, E. Ryabinkin, Y. Ryabov, A. Rybicki, S. Saarinen, S. Sadhu, S. Sadovsky, K. Šafařík, B. Sahlmuller, P. Sahoo, R. Sahoo, S. Sahoo, P. K. Sahu, J. Saini, S. Sakai, M. A. Saleh, J. Salzwedel, S. Sambyal, V. Samsonov, L. Šándor, A. Sandoval, M. Sano, D. Sarkar, N. Sarkar, P. Sarma, E. Scapparone, F. Scarlassara, C. Schiaua, R. Schicker, C. Schmidt, H. R. Schmidt, M. Schmidt, S. Schuchmann, J. Schukraft, M. Schulc, Y. Schutz, K. Schwarz, K. Schweda, G. Scioli, E. Scomparin, R. Scott, M. Šefčík, J. E. Seger, Y. Sekiguchi, D. Sekihata, I. Selyuzhenkov, K. Senosi, S. Senyukov, E. Serradilla, A. Sevcenco, A. Shabanov, A. Shabetai, O. Shadura, R. Shahoyan, M. I. Shahzad, A. Shangaraev, A. Sharma, M. Sharma, M. Sharma, N. Sharma, A. I. Sheikh, K. Shigaki, Q. Shou, K. Shtejer, Y. Sibiriak, S. Siddhanta, K. M. Sielewicz, T. Siemiarczuk, D. Silvermyr, C. Silvestre, G. Simatovic, G. Simonetti, R. Singaraju, R. Singh, S. Singha, V. Singhal, B. C. Sinha, T. Sinha, B. Sitar, M. Sitta, T. B. Skaali, M. Slupecki, N. Smirnov, R. J. M. Snellings, T. W. Snellman, J. Song, M. Song, Z. Song, F. Soramel, S. Sorensen, R. D. de Souza, F. Sozzi, M. Spacek, E. Spiriti, I. Sputowska, M. Spyropoulou-Stassinaki, J. Stachel, I. Stan, P. Stankus, E. Stenlund, G. Steyn, J. H. Stiller, D. Stocco, P. Strmen, A. A. P. Suaide, T. Sugitate, C. Suire, M. Suleymanov, M. Suljic, R. Sultanov, M. Šumbera, S. Sumowidagdo, A. Szabo, I. Szarka, A. Szczepankiewicz, M. Szymanski, U. Tabassam, J. Takahashi, G. J. Tambave, N. Tanaka, M. Tarhini, M. Tariq, M. G. Tarzila, A. Tauro, G. Tejeda Muñoz, A. Telesca, K. Terasaki, C. Terrevoli, B. Teyssier, J. Thäder, D. Thakur, D. Thomas, R. Tieulent, A. Tikhonov, A. R. Timmins, A. Toia, S. Trogolo, G. Trombetta, V. Trubnikov, W. H. Trzaska, T. Tsuji, A. Tumkin, R. Turrisi, T. S. Tveter, K. Ullaland, A. Uras, G. L. Usai, A. Utrobicic, M. Vala, L. Valencia Palomo, S. Vallero, J. Van Der Maarel, J. W. Van Hoorne, M. van Leeuwen, T. Vanat, P. Vande Vyvre, D. Varga, A. Vargas, M. Vargyas, R. Varma, M. Vasileiou, A. Vasiliev, A. Vauthier, O. Vázquez Doce, V. Vechernin, A. M. Veen, M. Veldhoen, A. Velure, E. Vercellin, S. Vergara Limón, R. Vernet, M. Verweij, L. Vickovic, J. Viinikainen, Z. Vilakazi, O. Villalobos Baillie, A. Villatoro Tello, A. Vinogradov, L. Vinogradov, Y. Vinogradov, T. Virgili, V. Vislavicius, Y. P. Viyogi, A. Vodopyanov, M. A. Völkl, K. Voloshin, S. A. Voloshin, G. Volpe, B. von Haller, I. Vorobyev, D. Vranic, J. Vrláková, B. Vulpescu, B. Wagner, J. Wagner, H. Wang, M. Wang, D. Watanabe, Y. Watanabe, M. Weber, S. G. Weber, D. F. Weiser, J. P. Wessels, U. Westerhoff, A. M. Whitehead, J. Wiechula, J. Wikne, G. Wilk, J. Wilkinson, M. C. S. Williams, B. Windelband, M. Winn, P. Yang, S. Yano, Z. Yasin, Z. Yin, H. Yokoyama, I.-K. Yoo, J. H. Yoon, V. Yurchenko, A. Zaborowska, V. Zaccolo, A. Zaman, C. Zampolli, H. J. C. Zanoli, S. Zaporozhets, N. Zardoshti, A. Zarochentsev, P. Závada, N. Zaviyalov, H. Zbroszczyk, I. S. Zgura, M. Zhalov, H. Zhang, X. Zhang, Y. Zhang, C. Zhang, Z. Zhang, C. Zhao, N. Zhigareva, D. Zhou, Y. Zhou, Z. Zhou, H. Zhu, J. Zhu, A. Zichichi, A. Zimmermann, M. B. Zimmermann, G. Zinovjev, M. Zyzak

**Affiliations:** 1 A.I. Alikhanyan National Science Laboratory (Yerevan Physics Institute) Foundation, Yerevan, Armenia; 2 Benemérita Universidad Autónoma de Puebla, Puebla, Mexico; 3 Bogolyubov Institute for Theoretical Physics, Kiev, Ukraine; 4 Department of Physics, Centre for Astroparticle Physics and Space Science (CAPSS), Bose Institute, Kolkata, India; 5 Budker Institute for Nuclear Physics, Novosibirsk, Russia; 6 California Polytechnic State University, San Luis Obispo, CA USA; 7 Central China Normal University, Wuhan, China; 8 Centre de Calcul de l’IN2P3, Villeurbanne, France; 9 Centro de Aplicaciones Tecnológicas y Desarrollo Nuclear (CEADEN), Havana, Cuba; 10 Centro de Investigaciones Energéticas Medioambientales y Tecnológicas (CIEMAT), Madrid, Spain; 11 Centro de Investigación y de Estudios Avanzados (CINVESTAV), Mexico City and Mérida, Mexico; 12 Centro Fermi-Museo Storico della Fisica e Centro Studi e Ricerche “Enrico Fermi”, Rome, Italy; 13 Chicago State University, Chicago, IL USA; 14 China Institute of Atomic Energy, Beijing, China; 15 Commissariat à l’Energie Atomique, IRFU, Saclay, France; 16 COMSATS Institute of Information Technology (CIIT), Islamabad, Pakistan; 17 Departamento de Física de Partículas and IGFAE, Universidad de Santiago de Compostela, Santiago de Compostela, Spain; 18 Department of Physics and Technology, University of Bergen, Mons, Norway; 19 Department of Physics, Aligarh Muslim University, Aligarh, India; 20 Department of Physics, Ohio State University, Columbus, OH USA; 21 Department of Physics, Sejong University, Seoul, South Korea; 22 Department of Physics, University of Oslo, Oslo, Norway; 23 Dipartimento di Fisica dell’Università ‘La Sapienza’ and Sezione INFN, Rome, Italy; 24 Dipartimento di Fisica dell’Università and Sezione INFN, Cagliari, Italy; 25 Dipartimento di Fisica dell’Università and Sezione INFN, Trieste, Italy; 26 Dipartimento di Fisica dell’Università and Sezione INFN, Turin, Italy; 27 Dipartimento di Fisica e Astronomia dell’Università and Sezione INFN, Bologna, Italy; 28 Dipartimento di Fisica e Astronomia dell’Università and Sezione INFN, Catania, Italy; 29 Dipartimento di Fisica e Astronomia dell’Università and Sezione INFN, Padua, Italy; 30 Dipartimento di Fisica ‘E.R. Caianiello’ dell’Università and Gruppo Collegato INFN, Salerno, Italy; 31 Dipartimento di Scienze e Innovazione Tecnologica dell’Università del Piemonte Orientale and Gruppo Collegato INFN, Alessandria, Italy; 32 Dipartimento Interateneo di Fisica ‘M. Merlin’ and Sezione INFN, Bari, Italy; 33 Division of Experimental High Energy Physics, University of Lund, Lund, Sweden; 34 Eberhard Karls Universität Tübingen, Tübingen, Germany; 35 European Organization for Nuclear Research (CERN), Geneva, Switzerland; 36 Excellence Cluster Universe, Technische Universität München, Munich, Germany; 37 Faculty of Engineering, Bergen University College, Mons, Norway; 38 Faculty of Mathematics, Physics and Informatics, Comenius University, Bratislava, Slovakia; 39 Faculty of Nuclear Sciences and Physical Engineering, Czech Technical University in Prague, Prague, Czech Republic; 40 Faculty of Science, P.J. Šafárik University, Kosice, Slovakia; 41 Faculty of Technology, Buskerud and Vestfold University College, Vestfold, Norway; 42 Frankfurt Institute for Advanced Studies, Johann Wolfgang Goethe-Universität Frankfurt, Frankfurt, Germany; 43 Gangneung-Wonju National University, Gangneung, South Korea; 44 Department of Physics, Gauhati University, Guwahati, India; 45 Helsinki Institute of Physics (HIP), Helsinki, Finland; 46 Hiroshima University, Hiroshima, Japan; 47 Indian Institute of Technology Bombay (IIT), Mumbai, India; 48 Indian Institute of Technology Indore (IITI), Indore, India; 49 Indonesian Institute of Sciences, Jakarta, Indonesia; 50 Inha University, Inchon, South Korea; 51 Institut de Physique Nucléaire d’Orsay (IPNO), Université Paris-Sud, CNRS-IN2P3, Orsay, France; 52 Institut für Informatik, Johann Wolfgang Goethe-Universität Frankfurt, Frankfurt, Germany; 53 Institut für Kernphysik, Johann Wolfgang Goethe-Universität Frankfurt, Frankfurt, Germany; 54 Institut für Kernphysik, Westfälische Wilhelms-Universität Münster, Münster, Germany; 55 Institut Pluridisciplinaire Hubert Curien (IPHC), Université de Strasbourg, CNRS-IN2P3, Strasbourg, France; 56 Institute for Nuclear Research, Academy of Sciences, Moscow, Russia; 57 Institute for Subatomic Physics of Utrecht University, Utrecht, The Netherlands; 58 Institute for Theoretical and Experimental Physics, Moscow, Russia; 59 Institute of Experimental Physics, Slovak Academy of Sciences, Kosice, Slovakia; 60 Institute of Physics, Academy of Sciences of the Czech Republic, Prague, Czech Republic; 61 Institute of Physics, Bhubaneswar, India; 62 Institute of Space Science (ISS), Bucharest, Romania; 63 Instituto de Ciencias Nucleares, Universidad Nacional Autónoma de México, Mexico City, Mexico; 64 Instituto de Física, Universidad Nacional Autónoma de México, Mexico City, Mexico; 65 iThemba LABS, National Research Foundation, Somerset West, South Africa; 66 Joint Institute for Nuclear Research (JINR), Dubna, Russia; 67 Konkuk University, Seoul, South Korea; 68 Korea Institute of Science and Technology Information, Daejeon, South Korea; 69 KTO Karatay University, Konya, Turkey; 70 Laboratoire de Physique Corpusculaire (LPC), Clermont Université, Université Blaise Pascal, CNRS-IN2P3, Clermont-Ferrand, France; 71 Laboratoire de Physique Subatomique et de Cosmologie, Université Grenoble-Alpes, CNRS-IN2P3, Grenoble, France; 72 Laboratori Nazionali di Frascati, INFN, Frascati, Italy; 73 Laboratori Nazionali di Legnaro, INFN, Legnaro, Italy; 74 Lawrence Berkeley National Laboratory, Berkeley, CA USA; 75 Moscow Engineering Physics Institute, Moscow, Russia; 76 Nagasaki Institute of Applied Science, Nagasaki, Japan; 77 National Centre for Nuclear Studies, Warsaw, Poland; 78 National Institute for Physics and Nuclear Engineering, Bucharest, Romania; 79 National Institute of Science Education and Research, Bhubaneswar, India; 80 National Research Centre Kurchatov Institute, Moscow, Russia; 81 Niels Bohr Institute, University of Copenhagen, Copenhagen, Denmark; 82 Nikhef, Nationaal instituut voor subatomaire fysica, Amsterdam, The Netherlands; 83 Nuclear Physics Group, STFC Daresbury Laboratory, Daresbury, UK; 84 Nuclear Physics Institute, Academy of Sciences of the Czech Republic, Řež u Prahy, Czech Republic; 85 Oak Ridge National Laboratory, Oak Ridge, TN USA; 86 Petersburg Nuclear Physics Institute, Gatchina, Russia; 87 Physics Department, Creighton University, Omaha, NE USA; 88 Physics Department, Panjab University, Chandigarh, India; 89 Physics Department, University of Athens, Athens, Greece; 90 Physics Department, University of Cape Town, Cape Town, South Africa; 91 Physics Department, University of Jammu, Jammu, India; 92 Physics Department, University of Rajasthan, Jaipur, India; 93 Physik Department, Technische Universität München, Munich, Germany; 94 Physikalisches Institut, Ruprecht-Karls-Universität Heidelberg, Heidelberg, Germany; 95 Purdue University, West Lafayette, IN USA; 96 Pusan National University, Pusan, South Korea; 97 Research Division and ExtreMe Matter Institute EMMI, GSI Helmholtzzentrum für Schwerionenforschung, Darmstadt, Germany; 98 Rudjer Bošković Institute, Zagreb, Croatia; 99 Russian Federal Nuclear Center (VNIIEF), Sarov, Russia; 100 Saha Institute of Nuclear Physics, Kolkata, India; 101 School of Physics and Astronomy, University of Birmingham, Birmingham, UK; 102 Sección Física, Departamento de Ciencias, Pontificia Universidad Católica del Perú, Lima, Peru; 103 Sezione INFN, Bari, Italy; 104 Sezione INFN, Bologna, Italy; 105 Sezione INFN, Cagliari, Italy; 106 Sezione INFN, Catania, Italy; 107 Sezione INFN, Padua, Italy; 108 Sezione INFN, Rome, Italy; 109 Sezione INFN, Trieste, Italy; 110 Sezione INFN, Turin, Italy; 111 SSC IHEP of NRC Kurchatov institute, Protvino, Russia; 112 Stefan Meyer Institut für Subatomare Physik (SMI), Vienna, Austria; 113 SUBATECH, Ecole des Mines de Nantes, Université de Nantes, CNRS-IN2P3, Nantes, France; 114 Suranaree University of Technology, Nakhon Ratchasima, Thailand; 115 Technical University of Košice, Kosice, Slovakia; 116 Technical University of Split FESB, Split, Croatia; 117 The Henryk Niewodniczanski Institute of Nuclear Physics, Polish Academy of Sciences, Cracow, Poland; 118 Physics Department, The University of Texas at Austin, Austin, TX USA; 119 Universidad Autónoma de Sinaloa, Culiacán, Mexico; 120 Universidade de São Paulo (USP), São Paulo, Brazil; 121 Universidade Estadual de Campinas (UNICAMP), Campinas, Brazil; 122 University of Houston, Houston, TX USA; 123 University of Jyväskylä, Jyväskylä, Finland; 124 University of Liverpool, Liverpool, UK; 125 University of Tennessee, Knoxville, TN USA; 126 University of the Witwatersrand, Johannesburg, South Africa; 127 University of Tokyo, Tokyo, Japan; 128 University of Tsukuba, Tsukuba, Japan; 129 University of Zagreb, Zagreb, Croatia; 130 Université de Lyon, Université Lyon 1, CNRS/IN2P3, IPN-Lyon, Villeurbanne, France; 131 Università di Brescia, Brescia, Italy; 132 V. Fock Institute for Physics, St. Petersburg State University, St. Petersburg, Russia; 133 Variable Energy Cyclotron Centre, Kolkata, India; 134 Warsaw University of Technology, Warsaw, Poland; 135 Wayne State University, Detroit, MI USA; 136 Wigner Research Centre for Physics, Hungarian Academy of Sciences, Budapest, Hungary; 137 Yale University, New Haven, CT USA; 138 Yonsei University, Seoul, South Korea; 139 Zentrum für Technologietransfer und Telekommunikation (ZTT), Fachhochschule Worms, Worms, Germany; 140 CERN, Geneva, Switzerland

## Abstract

Measurements of charged jet production as a function of centrality are presented for  p–Pb  collisions recorded at $\sqrt{s_\mathrm {NN}}= 5.02$ TeV with the ALICE detector. Centrality classes are determined via the energy deposit in neutron calorimeters at zero degree, close to the beam direction, to minimise dynamical biases of the selection. The corresponding number of participants or binary nucleon–nucleon collisions is determined based on the particle production in the Pb-going rapidity region. Jets have been reconstructed in the central rapidity region from charged particles with the anti-$k_\mathrm {T}$ algorithm for resolution parameters $R = 0.2$ and $R = 0.4$ in the transverse momentum range 20 to 120 GeV/*c*. The reconstructed jet momentum and yields have been corrected for detector effects and underlying-event background. In the five centrality bins considered, the charged jet production in  p–Pb   collisions is consistent with the production expected from binary scaling from pp collisions. The ratio of jet yields reconstructed with the two different resolution parameters is also independent of the centrality selection, demonstrating the absence of major modifications of the radial jet structure in the reported centrality classes.

## Introduction

The measurement of benchmark processes in proton–nucleus collisions plays a crucial role for the interpretation of nucleus–nucleus collision data, where one expects to create a system with high temperature in which the elementary constituents of hadronic matter, quarks and gluons, are deconfined for a short time: the quark-gluon plasma (QGP) [1]. Proton–lead collisions are important to investigate cold nuclear initial and final state effects, in particular to disentangle them from effects of the hot medium created in the final state of  Pb–Pb collisions [2].

The study of hard parton scatterings and their subsequent fragmentation via reconstructed jets plays a crucial role in the characterisation of the hot and dense medium produced in  Pb–Pb collisions while jet measurements in  p–Pb   and $\mathrm{pp}$ collisions provide allow to constrain the impact of cold nuclear matter effects in heavy-ion collisions. In the initial state, the nuclear parton distribution functions can be modified with respect to the quark and gluon distributions in free nucleons, e.g. via shadowing effects and gluon saturation [2, 3]. In addition, jet production may be influenced, already in  p–Pb   collisions, by multiple scattering of partons and hadronic re-interaction in the initial and final state [4, 5].

In the absence of any modification in the initial state, the partonic scattering rate in nuclear collisions compared to $\mathrm{pp}$ collisions is expected to increase linearly with the average number of binary nucleon–nucleon collisions $\langle N_\mathrm {coll}\rangle$. This motivates the definition of the nuclear modification factor $R_\mathrm {pPb}$, as the ratio of particle or jet transverse momentum ($p_\mathrm {T}$) spectra in nuclear collisions to those in $\mathrm{pp}$ collisions scaled by $\langle N_\mathrm {coll}\rangle$.

In heavy-ion collisions at the LHC, binary ($N_\mathrm {coll}$) scaling is found to hold for probes that do not interact strongly, i.e. isolated prompt photons [6] and electroweak bosons [7, 8]. On the contrary, the yields of hadrons and jets in central  Pb–Pb collisions are strongly modified compared to the scaling assumptions. For hadrons, the yield is suppressed by up to a factor of seven at $p_\mathrm {T}\approx 6$ GeV / *c*, approaching a factor of two at high $p_\mathrm {T}$ ($\gtrsim$30 GeV/*c*) [9–11]. A similar suppression is observed for jets [12–16]. This observation, known as jet quenching, is attributed to the formation of a QGP in the collision, where the hard scattered partons radiate gluons due to strong interaction with the medium, as first predicted in [17, 18].

In minimum bias  p–Pb  collisions at $\sqrt{s_\mathrm {NN}}= 5.02$ TeV the production of unidentified charged particles [19–22] and jets [23–25] is consistent with the absence of a strong final state suppression. However, multiplicity dependent studies in  p–Pb  collisions on the production of low-$p_\mathrm {T}$ identified particles and long range correlations [26–29] show similar features as measured in  Pb–Pb collisions, where they are attributed to the collective behaviour following the creation of a QGP. These features in  p–Pb   collisions become more pronounced for higher multiplicity events, which in  Pb–Pb are commonly associated with more central collisions or higher initial energy density.

The measurement of jets, compared to single charged hadrons, tests the parton fragmentation beyond the leading particle with the inclusion of large-angle and low-$p_\mathrm {T}$ fragments. Thus jets are potentially sensitive to centrality-dependent modifications of low-$p_\mathrm {T}$ fragments.

This work extends the analysis of the charged jet production in minimum bias  p–Pb  collisions recorded with the ALICE detector at $\sqrt{s_\mathrm {NN}}= 5.02$ TeV to a centrality-differential study for jet resolution parameters $R = 0.2$ and 0.4 in the $p_\mathrm {T}$ range from 20 to 120 GeV/*c* [25]. Section 2 describes the event and track selection, the centrality determination, as well as the jet reconstruction, the corrections for uncorrelated background contributing to the jet momentum [15, 30, 31] and the corrections for detector effects. The impact of different centrality selections on the nuclear modification factor has been studied in detail in [32]. We estimate the centrality using zero-degree neutral energy and the charged particle multiplicity measured by scintillator array detectors at rapidities along the direction of the Pb beam to determine $N_\mathrm {coll}$. The correction procedures specific to the centrality-dependent jet measurement are discussed in detail. Section 3 introduces the three main observables: the centrality-dependent jet production cross section, the nuclear modification factor, and ratio of jet cross sections for two different resolution parameters. Systematic uncertainties are discussed in Sect. 4 and results are presented in Sect. 5.

## Data analysis

### Event selection

The data used for this analysis were collected with the ALICE detector [33] during the  p–Pb  run of the LHC at $\sqrt{s_\mathrm {NN}} = 5.02 \mathrm{TeV}$ at the beginning of 2013. The ALICE experimental setup and its performance during the LHC Run 1 are described in detail in [33, 34].

For the analysis presented in this paper, the main detectors used for event and centrality selection are two scintillator detectors (V0A and V0C), covering the pseudo-rapidity range of $2.8 < \eta _\mathrm {lab}< 5.1$ and $-3.7 < \eta _\mathrm {lab} < -1.7$, respectively [35], and the Zero Degree Calorimeters (ZDCs), composed of two sets of neutron (ZNA and ZNC) and proton calorimeters (ZPA and ZPC) located at a distance $\pm 112.5$ m from the interaction point. Here and in the following $\eta _\mathrm {lab}$ denotes the pseudo-rapidity in the ALICE laboratory frame.

The minimum bias trigger used in  p–Pb  collisions requires signal coincidence in the V0A and V0C scintillators. In addition, offline selections on timing and vertex-quality are used to remove events with multiple interactions within the same bunch crossing and (pile-up) and background events, such as beam-gas interactions. The event sample used for the analysis presented in this manuscript was collected exclusively in the beam configuration where the proton travels towards negative $\eta _\mathrm {lab}$ (from V0A to V0C). The nucleon–nucleon center-of-mass system moves in the direction of the proton beam corresponding to a rapidity of $y_\mathrm {NN} = -0.465$.

A van der Meer scan was performed to measure the visible cross section for the trigger and beam configuration used in this analysis: $\sigma _\mathrm {V0} = 2.09 \pm 0.07$ b [36]. Studies with Monte Carlo simulations show that the sample collected in the configuration explained above consists mainly of non-single diffractive (NSD) interactions and a negligible contribution from single diffractive and electromagnetic interactions (see [37] for details). The trigger is not fully efficient for NSD events and the inefficiency is observed mainly for events without a reconstructed vertex, i.e. with no particles produced at central rapidity. Given the fraction of events without a reconstructed vertex in the data the corresponding inefficiency for NSD events is estimated to ($2.2~\pm ~3.1$) %. This inefficiency is expected to mainly affect the most peripheral centrality class. Following the prescriptions of [32], centrality classes are defined as percentiles of the visible cross section and are not corrected for trigger efficiency.

The further analysis requires a reconstructed vertex, in addition to the minimum bias trigger selection. The fraction of events with a reconstructed vertex is 98.3 % for minimum bias events and depends on the centrality class. In the analysis events with a reconstructed vertex $|z| > 10~\mathrm {cm}$ along the beam axis are rejected. In total, about $96\cdot 10^6$ events, corresponding to an integrated luminosity of 46 $\upmu$b${}^{-1}$, are used for the analysis and classified into five centrality classes

### Centrality determination

Centrality classes can be defined by dividing the multiplicity distribution measured in a certain pseudo-rapidity interval into fractions of the cross section, with the highest multiplicities corresponding to the most central collisions (smallest impact parameter *b*). The corresponding number of participants, as well as $N_\mathrm {coll}$ and *b*, can be estimated with a Glauber model [38], e.g. by fitting the measured multiplicity distribution with the $N_\mathrm {part}$ distribution from the model, convoluted with a Negative Binomial Distribution (NBD). Details on this procedure for  Pb–Pb and  p–Pb  collisions in ALICE are found in [32, 39], respectively.

In  p–*A* collisions centrality selection is susceptible to a variety of biases. In general, relative fluctuations of $N_\mathrm {part}$ and of event multiplicity are large, due to their small numerical value, in  p–Pb  collisions [32] $\langle N_\mathrm {part}\rangle = \langle N_\mathrm {coll}\rangle + 1 = 7.9 \pm 0.6$ and $\frac{\mathrm {d}N_\mathrm {ch}}{\mathrm {d}\eta } = 16.81 \pm 0.71$, respectively. Using either of these quantities to define centrality, in the Glauber model or the in experimental method, already introduces a bias compared to a purely geometrical selection based on the impact parameter *b*.

In addition, a kinematic bias exists for events containing high-$p_\mathrm {T}$ particles, originating from parton fragmentation as discussed above. The contribution of these jet fragments to the overall multiplicity rises with the jet energy and thus can introduce a trivial correlation between the multiplicity and presence of a high-$p_\mathrm {T}$ particle, and a selection on multiplicity will bias the jet population. High multiplicity events are more likely created in collisions with multiple-parton interactions, which can lead to a nuclear modification factor larger than unity. On the contrary, the selection of low multiplicity (peripheral) events can pose an effective veto on hard processes, which would lead to a nuclear modification factor smaller than unity. As shown in [32] the observed suppression and enhancement for charged particles in bins of multiplicity with respect to the binary scaling assumption can be explained by this selection bias alone. The bias can be fully reproduced by an independent superposition of simulated $\mathrm{pp}$ events and the farther the centrality estimator is separated in rapidity from the measurement region at mid-rapidity, the smaller the bias. We do not repeat the analysis for the centrality estimators with known biases here.

In this work, centrality classification is based solely on the zero-degree energy measured in the lead-going neutron detector ZNA, since it is expected to have only a small dynamical selection bias. However, the ZNA signal cannot be related directly to the produced multiplicity for the $N_\mathrm {coll}$ determination via NBD. As discussed in detail in [32] an alternative hybrid approach is used to connect the centrality selection based on the ZNA signal to another $N_\mathrm {coll}$ determination via the charged particle multiplicity in the lead-going direction measured with the V0A ($\langle N_\mathrm {coll}\rangle _c^\mathrm {Pb-side}$). This approach assumes that the V0 signal is proportional to the number of wounded lead (target) nucleons ($N_\mathrm {part}^\mathrm {target} = N_\mathrm {part}- 1 = N_\mathrm {coll}$). The average number of collisions for a given centrality, selected with the ZNA, is then given by scaling the minimum bias value $\langle N_\mathrm {coll}\rangle _\mathrm {MB} = 6.9$ with the ratio of the average raw signal *S* of the innermost ring of the V0A:

1 $$\begin{aligned} \langle N_\mathrm {coll}^\mathrm {Pb-side} \rangle _c = \langle N_\mathrm {coll}\rangle _\mathrm {MB} \cdot \frac{\langle S \rangle _c}{\langle S \rangle _\mathrm {MB}}. \end{aligned}$$

The values of $N_\mathrm {coll}$ obtained with this method are shown in Table 1 for different ZNA centrality classes [32].

**Table 1:** Average $N_\mathrm {coll}$ values for centrality classes selected with the ZNA determined with the hybrid approach ($N_\mathrm {coll}^\mathrm {Pb-side}$) [32], as well as moments of the background density and background fluctuation distributions shown in Fig. 1 (negligible statistical uncertainty)

ZNA centrality class (%) of visible cross section	$\langle N_\mathrm {coll}^\mathrm {Pb-side}\rangle$	$\rho$ (GeV/*c*)	$\sigma (\rho )$ (GeV/*c*)	$\sigma (\delta p_\mathrm {T,\,ch}) (R = 0.4)$ (GeV/*c*)
0–20	12.1$~\pm ~$1.0	1.60	1.17	1.43
20–40	9.6$~\pm ~$0.8	1.27	1.04	1.30
40–60	6.7$~\pm ~$0.5	0.88	0.84	1.11
60–80	4.0$~\pm ~$0.3	0.70	0.52	0.90
80–100	2.1$~\pm ~$0.3	0.26	0.37	0.71
Minimum bias (0–100)	6.9$~\pm ~$0.6	0.98	1.02	0.91

### Jet reconstruction and event-by-event corrections

The reported measurements are performed using *charged jets*, clustered starting from charged particles only, as described in [15, 25, 40] for different collision systems. Charged particles are reconstructed using information from the Inner Tracking System (ITS) [41] and the Time Projection Chamber (TPC) which cover the full azimuth and $|\eta _\mathrm {lab}| < 0.9$ for tracks reconstructed with full length in the TPC [42].

The azimuthal distribution of high-quality tracks with reconstructed track points in the Silicon Pixel Detector (SPD), the two innermost layers of the ITS, is not completely uniform due to inefficient regions in the SPD. This can be compensated by considering in addition tracks *without* reconstructed points in the SPD. The additional tracks constitute approximately 4.3 % of the track sample used for analysis. For these tracks, the primary vertex is used as an additional constraint in the track fitting to improve the momentum resolution. This approach yields a uniform tracking efficiency within the acceptance, which is needed to avoid geometrical biases of the jet reconstruction algorithm caused by a non-uniform density of reconstructed tracks. The procedure is described first and in detail in the context of jet reconstruction with ALICE in  Pb–Pb collisions [15].

**Fig. 1:**
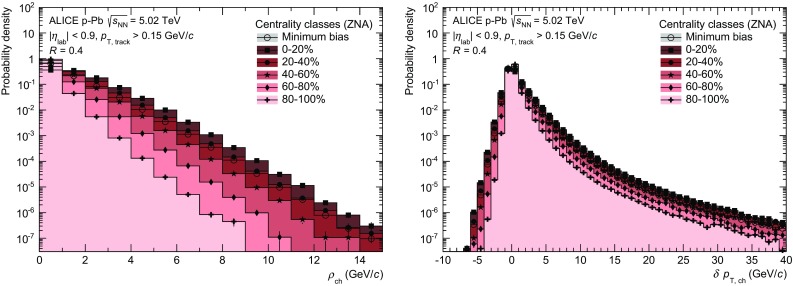
(Color online) *Left* Centrality dependence of the background momentum density $\rho _\mathrm {ch}$ determined with $k_\mathrm {T}$ jets and $R = 0.4$. Right: $\delta p_\mathrm {T,\,ch}$ distributions for different centralities obtained with random cones and $R = 0.4$

The anti-$k_\mathrm {T}$ algorithm from the FastJet package [43] is employed to reconstruct jets from these tracks using the $p_\mathrm {T}$ recombination scheme. The resolution parameters used in the present analysis are $R=0.2$ and $R=0.4$. Reconstructed jets are further corrected for contributions from the underlying event to the jet momentum as

2 $$\begin{aligned} p_\mathrm {T,\,ch\;jet} = p_\mathrm {T,\,ch\;jet}^\mathrm {raw} - A_\mathrm {ch\;jet} \cdot \rho _\mathrm {ch}, \end{aligned}$$

where $A_\mathrm {ch\;jet}$ is the area of the jet and $\rho _\mathrm {ch}$ the event-by-event background density [44]. The area is estimated by counting the so-called *ghost particles* in the jet. These are defined as particles with a finite area and vanishing momentum, which are distributed uniformly in the event and included in the jet reconstruction [45]. Their vanishing momentum ensures that the jet momentum is not influenced when they are included, while the number of ghost particles assigned to the jet provides a direct measure of its area. The background density $\rho _\mathrm {ch}$ is estimated via the median of the individual momentum densities of jets reconstructed with the $k_\mathrm {T}$ algorithm in the event

3 $$\begin{aligned} \rho _\mathrm {ch} = \mathrm {median} \left\{ \frac{p_{ \mathrm {T},\,k}}{A_k} \right\} \cdot C, \end{aligned}$$

where *k* runs over all reconstructed $k_\mathrm {T}$ jets with momentum $p_{\mathrm {T},\,i}$ and area $A_i$. Reconstructed $k_\mathrm {T}$ jets are commonly chosen for the estimate of the background density, since they provide a more robust sampling of low momentum particles. *C* is the occupancy correction factor, defined as

4 $$\begin{aligned} C = \frac{\sum _j {A_{j} }}{A_\mathrm{acc}}, \end{aligned}$$

where $A_j$ is the area of each $k_\mathrm {T}$ jet with at least one real track, i.e. excluding ghosts, and $A_\mathrm {acc}$ is the area of the charged-particle acceptance, namely $(2 \times 0.9) \times 2\pi$. The typical values for *C* range from 0.72 for most central collisions (0–20 %) to 0.15 for most peripheral collisions (80–100 %). This procedure takes into account the more sparse environment in  p–Pb  collisions compared to  Pb–Pb and is described in more detail in [25]. The probability distribution for $\rho _\mathrm {ch}$ for the five centrality classes and minimum bias is shown in Fig. 1 (left) and the mean and width of the distributions are given in Table 1. The event activity and thus the background density increases for more central collisions, though on average the background density is still two orders of magnitude smaller than in  Pb–Pb collisions where $\rho _\mathrm {ch}$ is ${\approx }140$ GeV/*c* for central collisions [31].

### Jet spectrum unfolding

Residual background fluctuations and instrumental effects can smear the jet $p_\mathrm {T}$. Their impact on the jet spectrum needs to be corrected on a statistical basis using unfolding, which is performed using the approach of Singular-Value-Decomposition (SVD) [46]. The response matrix employed in the unfolding is the combination of the (centrality-dependent) jet response to background fluctuations and the detector response. The general correction techniques are discussed in detail in the context of the minimum bias charged jet measurement in  p–Pb   [25].

Region-to-region fluctuations of the background density compared to the event median, contain purely statistical fluctuations of particle number and momentum and in addition also intra-event correlations, e.g. those characterised by the azimuthal anisotropy $v_2$ and higher harmonics, which induce additional variations of the local background density. The impact of these fluctuations on the jet momentum is determined by probing the transverse momentum density in randomly distributed cones in $(\eta ,\phi )$ and comparing it to the average background via [31]:

5 $$\begin{aligned} \delta p_\mathrm {T,\,ch}= \sum _\mathrm {i}{p_\mathrm {T,\,i}-\rho _\mathrm {ch}\cdot A}, ~~~ A = \pi R^2 \end{aligned}$$

where $p_\mathrm {T,\,i}$ is the transverse momentum of each track *i* inside a cone of radius *R*, where *R* corresponds to the resolution parameter in the jet reconstruction. $\rho _\mathrm {ch}$ is the background density, and *A* the area of the cone. The distribution of residuals, as defined by Eq. 5, is shown for different centralities in Fig. 1 (right). The corresponding widths are given in Table 1. The background fluctuations increase for more central events, which is expected from the general increase of statistical fluctuations ($\propto \sqrt{N}$) with the particle multiplicity. The $\delta p_\mathrm {T,\,ch}$ distributions measured for $R = 0.2$ and 0.4 are used in the unfolding procedure.

In addition to the background fluctuations the unfolding procedure takes into account the instrumental response. The dominating instrumental effects on the reconstructed jet spectrum are the single-particle tracking efficiency and momentum resolution. These effects are encoded in a response matrix, which is determined with a full detector simulation using PYTHIA6 [47] to generate jets and GEANT3 [48] for the transport through the ALICE setup. The detector response matrix links the jet momentum at the charged particle level to the one reconstructed from tracks after particle transport through the detector. No correction for the missing energy of neutral jet constituents is applied.

## Observables

### Jet production cross sections

The jet production cross sections $\frac{\mathrm {d}\sigma ^c}{\mathrm {d}p_\mathrm {T}}$, for different centralities *c*, are provided as fractions of the visible cross section $\sigma _\mathrm {V0}$. The fraction of the cross section is determined with the number of selected events in each centrality bin $N^c_\mathrm {ev}$ and takes into account the vertex reconstruction efficiency $\varepsilon ^c_\mathrm {vtx}$ determined for each centrality

6 $$\begin{aligned} \frac{\mathrm {d}\sigma ^c}{\mathrm {d}p_\mathrm {T}} = \frac{\varepsilon ^c_\mathrm {vtx}}{N^c_\mathrm {ev}} \frac{\mathrm {d}N}{\mathrm {d}p_\mathrm {T}} \cdot \sigma _\mathrm {V0} \cdot \frac{N^c_\mathrm {ev}}{N^\mathrm {MB}_\mathrm {ev}} = \frac{\varepsilon ^c_\mathrm {vtx}}{N^\mathrm {MB}_\mathrm {ev}} \frac{\mathrm {d}N}{\mathrm {d}p_\mathrm {T}} \cdot \sigma _\mathrm {V0}, \end{aligned}$$

where $\varepsilon ^c_\mathrm {vtx}$ decreases from 99.9 % for the most central selection (0–20 %) to 95.4 % in peripheral.

### Quantifying nuclear modification

The nuclear modification factor compares the $p_\mathrm {T}$-differential per-event yield, e.g. in  p–Pb  or  Pb–Pb collisions, to the differential yield in $\mathrm{pp}$ collisions at the same center-of-mass energy in order to quantify nuclear effects. Under the assumption that the jet or particle production at high $p_\mathrm {T}$ scales with the number of binary collisions, the nuclear modification factor is unity in the absence of nuclear effects.

In  p–Pb  collisions the jet population can be biased, depending on the centrality selection and $N_\mathrm {coll}$ determination, hence the nuclear modification factor may vary from unity even in the absence of nuclear effects as described in detail in Sect. 2.2 (see also [32]). To reflect this ambiguity the centrality-differential nuclear modification factor in  p–Pb   collisions is called $Q_\mathrm {pPb}$, instead of $R_\mathrm {pPb}$ as in the minimum bias case. $Q_\mathrm {pPb}$ is defined as

7 $$\begin{aligned} Q_\mathrm {pPb}= \frac{ \mathrm {d^2}N^{c}_\mathrm {pPb}/\mathrm {d}\eta \mathrm {d}p_\mathrm {T}}{\langle N_\mathrm {coll}^{c}\rangle \cdot \mathrm {d^2}N_\mathrm {pp}/\mathrm {d}\eta \mathrm {d}p_\mathrm {T}}. \end{aligned}$$

Here, $\langle N^{c}_\mathrm {coll}\rangle$ is number of binary collisions for centrality *c*, shown in Table 1.

For the construction of $Q_\mathrm {pPb}$, we use the same $\mathrm{pp}$ reference as for the study of charged jet production in minimum bias  p–Pb   collisions [25]. This reference has been determined from the ALICE charged jet measurement at 7 TeV [40] via scaling to the  p–Pb  center-of-mass energy and taking into account the rapidity shift of the colliding nucleons. The scaling behaviour of the charged jet spectra is determined based on pQCD calculations using the POWHEG framework [49] and PYTHIA parton shower (see [25] for details). This procedure fixes the normalisation based on the measured data at 7 TeV, while the evolution of the cross section with beam energy is calculated, taking into account all dependences implemented in POWHEG and PYTHIA, e.g. the larger fraction of quark initiated jets at lower collision energy.

### Jet production cross section ratio

The angular broadening or narrowing of the parton shower with respect to the original parton direction can have an impact on the jet production cross section determined with different resolution parameters. This can be tested via the ratio of cross sections or yields reconstructed with different radii, e.g. $R = 0.2$ and 0.4, in a common rapidity interval, here $|\eta _\mathrm {lab}| < 0.5$:

8 $$\begin{aligned} \mathscr {R}(0.2,\,0.4) = \frac{\mathrm {d}\sigma _\mathrm {pPb,\, R=0.2} / \mathrm {d}p_\mathrm {T}}{\mathrm {d}\sigma _\mathrm {pPb,\, R=0.4} / \mathrm {d}p_\mathrm {T}}. \end{aligned}$$

Consider for illustration the extreme scenario where all fragments are already contained within $R=0.2$. In this case the ratio would be unity. In addition, the statistical uncertainties between $R = 0.2$ and $R = 0.4$ would be fully correlated and they would cancel completely in the ratio, when the jets are reconstructed from the same data set. If the jets are less collimated, the ratio decreases and the statistical uncertainties cancel only partially. For the analysis presented in this paper, the conditional probability varies between 25 and 50 % for reconstructing a $R =0.2$ jet in the same $p_\mathrm {T}$-bin as a geometrically close $R = 0.4$ jet. This leads to a reduction of the statistical uncertainty on the ratio of about 5–10 % compared to the case of no correlation.

The measurement and comparison of fully corrected jet cross sections for different radii provides an observable sensitive to the radial redistribution of momentum that is also theoretically well defined [50]. Other observables that test the structure of jets, such as the fractional transverse momentum distribution of jet constituents in radial and longitudinal direction or jet-hadron correlations [10, 51–54], are potentially more sensitive to modified jet fragmentation in   p–Pb  and  Pb–Pb . However, in these cases the specific choices of jet reconstruction parameters, particle $p_\mathrm {T}$ thresholds and the treatment of background particles often limit the quantitative comparison between experimental observables and to theory calculations.

## Systematic uncertainties

The different sources of systematic uncertainties for the three observables presented in this paper are listed in Table 2 for 0–20 % and 60–80 % most central collisions.

**Table 2:** Summary of systematic uncertainties on the fully corrected jet spectrum, the corresponding nuclear modification factor, and the jet production cross section ratio in 0–20 % central and 60–80 % peripheral events for the resolution parameter $R=0.4$. The range of percentages provides the variation from the minimum to the maximum momentum in each centrality. For $R = 0.2$ only the combined uncertainty is provided for, the difference to $R = 0.4$ is mainly due to the smaller impact of the single particle efficiency for smaller radii

Observable	Jet cross section ($R = 0.4$)		$Q_\mathrm {pPb}$ ($R = 0.4$)		$\mathscr {R}$	
ZNA centrality class (%)	0–20	60–80	0–20	60–80	0–20	60–80
Single-particle efficiency (%)	10.2–14.0	10.0–12.7	4.9–6.3	4.9–6.4	2.0–2.0	1.8–4.7
Unfolding (%)	4.3	4.6	4.5	4.8	1.4	$-$3.1
Unfolding prior steepness (%)	0.9–7.0	0.3–3.6	1.1–7.2	0.8–4.0	0.7–1.4	0.3–2.2
Regularisation strength (%)	2.8–6.4	0.4–3.7	2.8–7.3	0.5–3.9	1.8–7.0	0.3–3.7
Minimum $p_\mathrm {T}$ cut-off (%)	3.7–9.2	0.6–2.9	4.1–9.8	1.7–3.8	2.2–0.8	0.5–1.8
Background estimate (%)	3.5–1.8	3.8–3.0	3.5–1.8	3.8–3.0	1.7–1.8	2.6–1.2
$\delta p_\mathrm {T,\,ch}$ estimate (%)	0.1–0.0	0.2–2.3	0.1–0.0	0.2–2.3	0.1–0.0	0.2–1.1
Combined uncertainty (%)	12.5–19.8	11.6–15.2	9.0–16.3	8.1–11.1	4.2–7.8	4.4–7.5
Combined uncertainty (R = 0.2) (%)	10.4–19.5	8.2–12.5	8.6–18.0	5.8–9.4	–	–
$\langle N_\mathrm{coll}^\mathrm{Pb-side}\rangle$ (%)	–	–	8.0	8.0	–	–
Visible cross section (%)	3.3	3.3	–	–	–	–
Reference scaling pp 7 TeV (%)	–	–	9.0	9.0	–	–
NSD selection efficiency p–Pb (%)	–	–	3.1	3.1	–	–
Combined scaling uncertainty (%)	–	–	12.4	12.4	–	–

The dominant source of uncertainty for the $p_\mathrm {T}$-differential jet production cross section is the uncertainty of the single-particle tracking efficiency that has a direct impact on the correction of the jet momentum in the unfolding, as discussed in Sect. 2.4. In  p–Pb  collisions, the single-particle efficiency is known with a relative uncertainty of 4 %, which is equivalent to a 4 % uncertainty on the jet momentum scale. To estimate the effect of the tracking efficiency uncertainty on the jet yield, the tracking efficiency is artificially lowered by randomly discarding the corresponding fraction of tracks (4 %) used as input for the jet finder. Depending on the shape of the spectrum, the uncertainty on the single-particle efficiency (jet momentum scale) translates into an uncertainty on the jet yield ranging from 8 to 15 %.

To estimate the effect of the single-particle efficiency on the   p–Pb  nuclear modification factor for jets, one has to consider that the uncertainty on the efficiency is partially correlated between the $\mathrm{pp}$ and  p–Pb  data set. The correction is determined with the same description of the ALICE detector in the Monte Carlo and for similar track quality cuts, but changes of detector conditions between run periods reduce the degree of correlation between the data sets. The uncorrelated uncertainty on the single-particle efficiency has been estimated to 2 % by varying the track quality cuts in data and simulations. Consequently, the resulting uncertainty for the nuclear modification factor is basically half the uncertainty due to the single particle efficiency in the jet spectrum (cf. Table 2). It was determined by discarding 2 % of the tracks in one of the two collision systems, as also described in [25].

Uncertainties introduced by the unfolding procedure, e.g. choice of unfolding method, prior, regularisation strength, and minimum $p_\mathrm {T}$ cut-off, are determined by varying those methods and parameters within reasonable boundaries. Bayesian [55, 56] and $\chi ^2$ [57] unfolding have been tested and compared to the default SVD unfolding to estimate the systematic uncertainty of the chosen method. The quality of the unfolded result is evaluated by inspecting the Pearson coefficients, where a large (anti-)correlation between neighbouring bins indicates that the regularisation is not optimal.

The overall uncertainty on the jet yield due to the background subtraction is estimated by comparing various background estimates: track-based and jet-based density estimates, as well as pseudo-rapidity-dependent corrections. The estimated uncertainty amounts to 3.8 % at low $p_\mathrm {T}$ and decreases for higher reconstructed jet momenta.

The main uncertainty related to the background fluctuation estimate is given by the choice of excluding reconstructed jets in the random cone sampling. While the probability of a jet to overlap with another jet in the event scales with $N_\mathrm {coll}-1$, it scales in the case of the random cone sampling with $N_\mathrm {coll}$. This can be emulated by rejecting a given fraction of cones overlapping with signal jets, which introduces an additional dependence on the definition of a signal jet. The resulting uncertainty due to the treatment of jet overlaps is of the order of 0.1 % and can be considered negligible.

In addition, several normalisation uncertainties need to be considered: the uncertainty on $N_\mathrm {coll}$ (8 % in the hybrid approach), on the visible cross section $\sigma _\mathrm {V0}$ (3.3 %) and from the assumptions made to obtain the scaled pp reference from 7 to 5 TeV (9 %).

Further details on the evaluation of the centrality-independent systematic uncertainties can be found in [25].

## Results

**Fig. 2:**
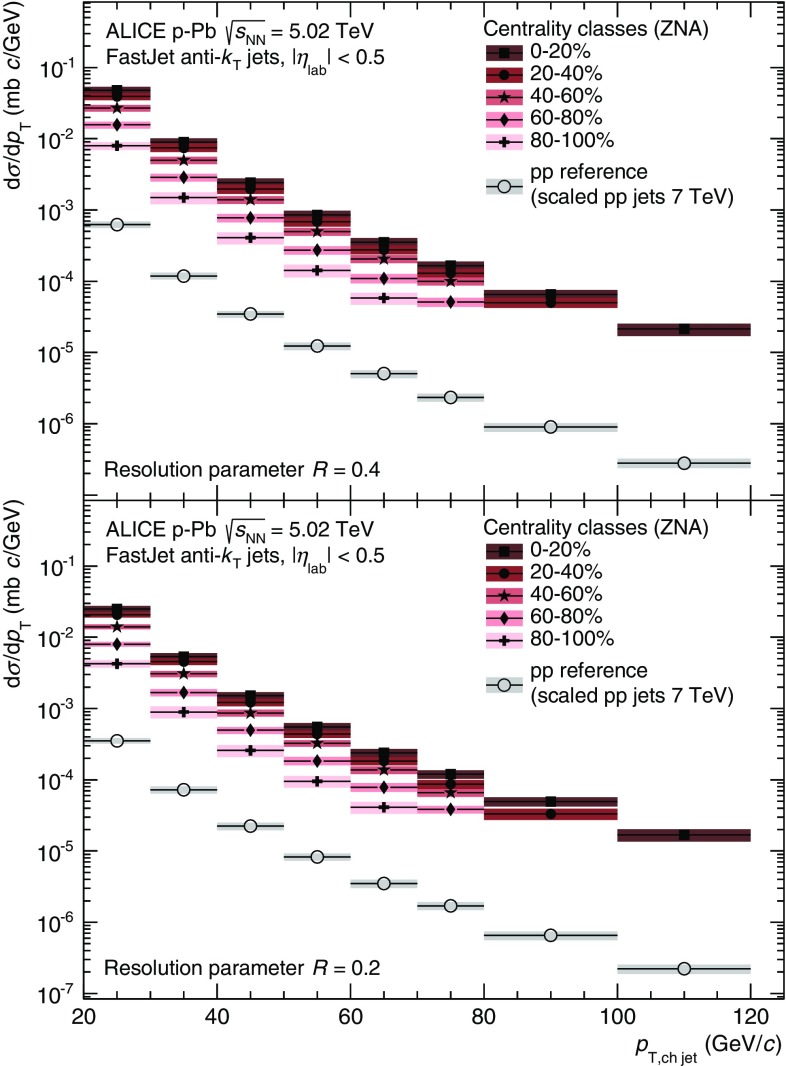
(Color online) $p_\mathrm {T}$-differential production cross sections of charged jet production in  p–Pb collisions at 5.02 TeV for several centrality classes. *Top* and *bottom* panels show the result for $R=0.4$ and $R=0.2$, respectively. In these and the following plots, the *coloured boxes* represent systematic uncertainties, the *error bars* represent statistical uncertainties. The overall normalisation uncertainty on the visible cross section is $3.3\,\%$ in  p–Pb . The corresponding reference $\mathrm{pp}$ spectrum is shown for both radii, it was obtained by scaling down the measured charged jets at 7 TeV to the reference energy

The $p_\mathrm {T}$-differential cross sections for jets reconstructed from charged particles for five centrality classes in  p–Pb collisions at $\sqrt{s_\mathrm {NN}}= 5.02$ TeV are shown in Fig. 2. For both resolution parameters, the measured yields are higher for more central collisions, as expected from the increase of the binary interactions (cf. Table 1). The $\mathrm{pp}$ reference at $\sqrt{s} = 5.02$ TeV is also shown. In addition to the increase in binary collisions the larger total cross section in  p–Pb compared to $\mathrm{pp}$ further separates the data from the two collision systems; by an additional factor of $20\,\% \cdot \sigma ^{\mathrm {pPb}}_\mathrm {V0}/\sigma ^\mathrm {pp}_\mathrm {inel} \approx 6$.

**Fig. 3:**
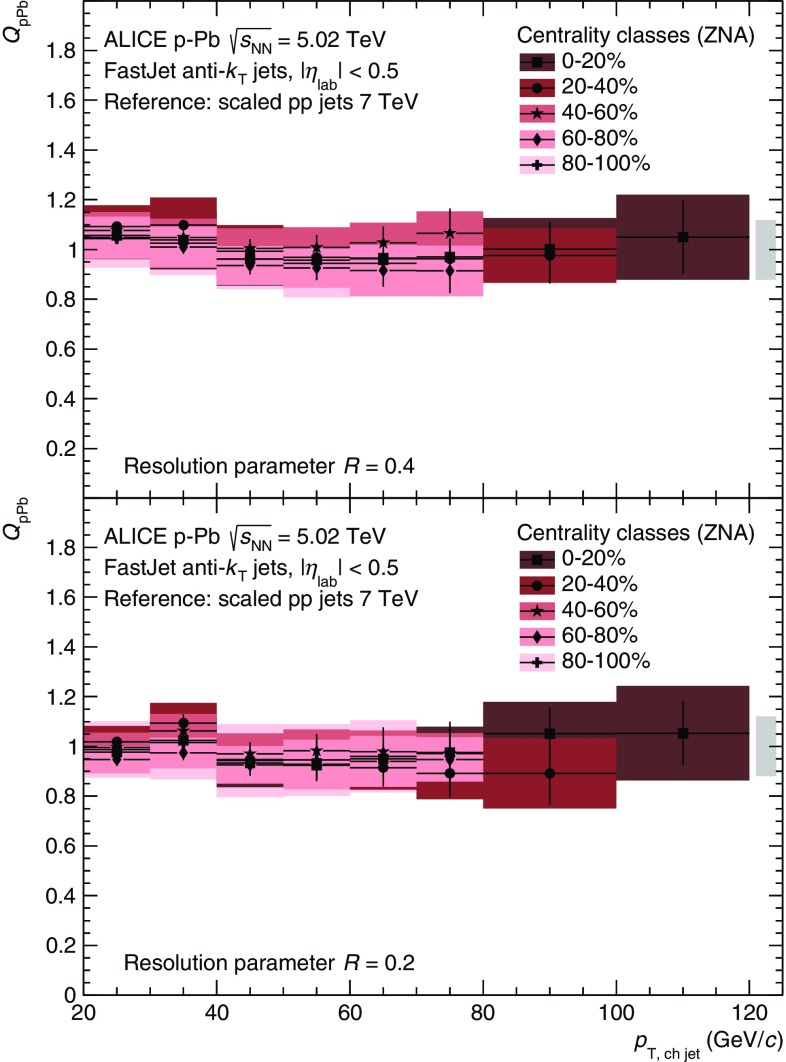
(Color online) Nuclear modification factors $Q_\mathrm {pPb}$ of charged jets for several centrality classes. $N_\mathrm {coll}$ has been determined with the hybrid model. *Top* and *bottom* panels show the result for $R=0.4$ and $R=0.2$, respectively. The combined global normalisation uncertainty from $N_\mathrm {coll}$, the measured pp cross section, and the reference scaling is indicated by the *box* around unity

The scaling behaviour of the  p–Pb spectra with respect to the pp reference is quantified by the nuclear modification factor $Q_\mathrm {pPb}$ (Eq.7). The nuclear modification factor with the hybrid approach, shown in Fig. 3, is compatible with unity for all centrality classes, indicating the absence of centrality-dependent nuclear effects on the jet yield in the kinematic regime probed by our measurement. This result is consistent with the measurement of single charged particles in  p–Pb collisions presented in [32], where the same hybrid approach is used.

For other centrality selections, closer to mid-rapidity, a separation of $Q_\mathrm {pPb}$ for jets is observed for the different centralities that is caused by dynamical biases of the selection, similar to the $Q_\mathrm {pPb}$ for charged particles. If we use e.g. the centrality selection based on the multiplicity in the V0A, $Q_\mathrm {pPb}$ decreases from about 1.2 in central to approximately 0.5 in peripheral collisions [58].

**Fig. 4:**
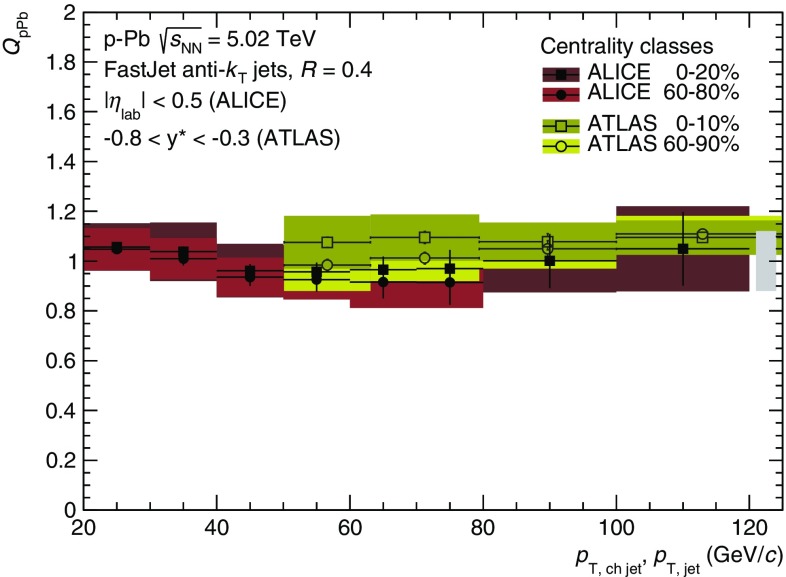
(Color online) Nuclear modification factor of charged jets compared to the nuclear modification factor for full jets as measured by the ATLAS collaboration [23]. Note that the underlying parton $p_\mathrm {T}$ for fixed reconstructed jet $p_\mathrm {T}$ is higher in the case of charged jets

The centrality dependence of full jet production in  p–Pb collisions, i.e. using charged and neutral jet fragments, has been reported by the ATLAS collaboration in [23] over a broad range of the center-of-mass rapidity ($y^{*}$) and transverse momentum. Centrality-dependent deviations of jet production have been found for large rapidities in the proton-going direction and $p_\mathrm {T, jet} \gtrsim 100$ GeV/*c*. In the nucleon–nucleon center-of-mass system as defined by ATLAS, our measurement in $\left| \eta _\mathrm {lab}\right| < 0.5$ corresponds to $-0.96 < y^{*} < -0.04$. As shown in Fig. 4, the measurement of the nuclear modification factor of charged jets in central and peripheral collisions is consistent with the full jet measurement of ATLAS, where the kinematical selection of jet momentum and rapidity overlap, note however that the underlying parton $p_\mathrm {T}$ at a given reconstructed $p_\mathrm {T}$ is higher for charged jets.

**Fig. 5:**
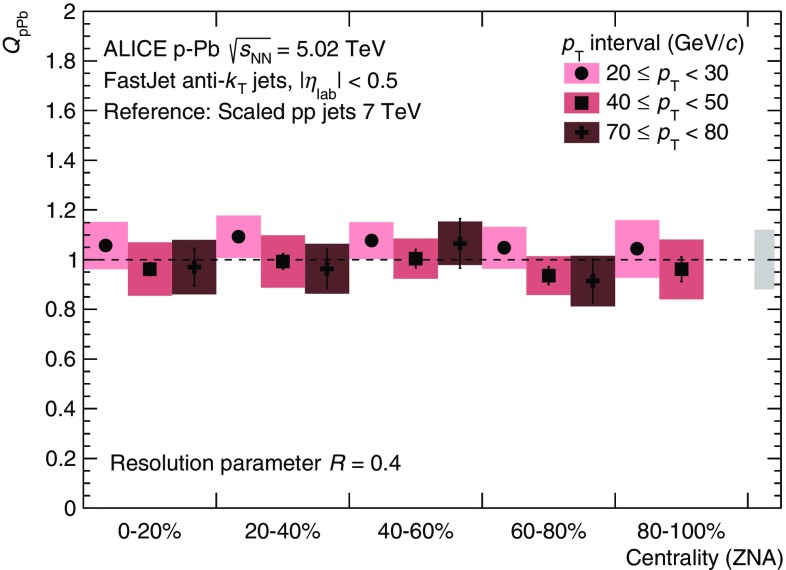
(Color online) Centrality evolution of $Q_\mathrm {pPb}$ for selected $p_\mathrm {T,\,ch\;jet}$-bins and $R = 0.4$

The centrality evolution for $Q_\mathrm {pPb}$ as measured by ALICE is shown for three $p_\mathrm {T}$-regions and $R = 0.4$ in Fig. 5. No significant variation is observed with centrality for a fixed $p_\mathrm {T}$ interval. The same holds for $R = 0.2$ (not shown).

Recently, the PHENIX collaboration reported on a centrality dependent modification of the jet yield in  d–Au collisions at $\sqrt{s_\mathrm {NN}}= 200$ GeV in the range of $20 < p_\mathrm {T}< 50~\text{ GeV }/c$ [59]: a suppression of 20 % in central events and corresponding enhancement in peripheral events is observed. Even when neglecting the impact of any possible biases in the centrality selection, the measurement of the nuclear modification at lower $\sqrt{s_\mathrm {NN}}$ cannot be directly compared to the measurements at LHC for two reasons. First, in case of a possible final state energy loss the scattered parton momentum is the relevant scale. Here, the nuclear modification factor at lower energies is more sensitive to energy loss, due to the steeper spectrum of scattered partons. Second, for initial state effects the nuclear modification should be compared in the probed Bjorken-*x*, which can be estimated at mid-rapidity to $x_\mathrm {T} \approx 2p_\mathrm {T}/\sqrt{s_\mathrm {NN}}$, and is at a given $p_\mathrm {T}$ approximately a factor of 25 smaller in  p–Pb  collisions at the LHC.

**Fig. 6:**
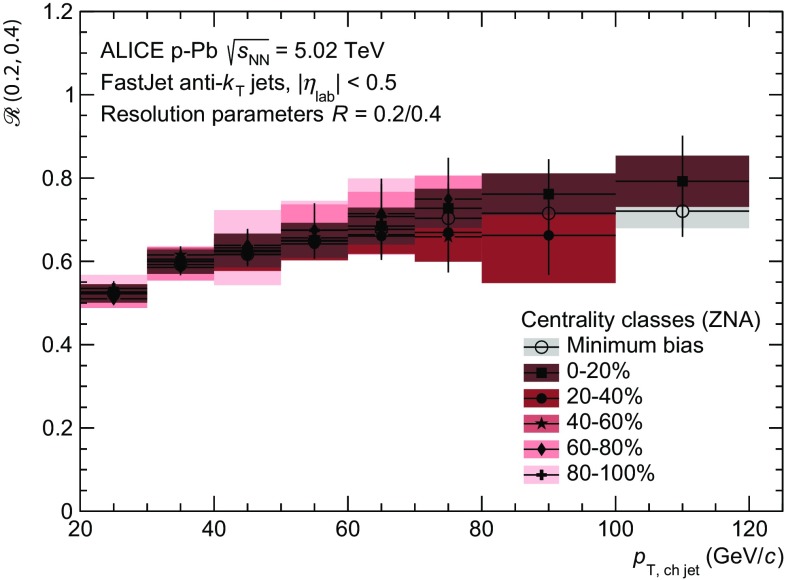
(Color online) Charged jet production cross section ratio for different resolution parameters as defined in Eq. 8. Different centrality classes are shown together with the result for minimum bias collisions. Note that the systematic uncertainties are partially correlated between centrality classes. The ratio for minimum collisions is compared in more detail to $\mathrm{pp}$ collisions at higher energy and NLO calculations at $\sqrt{s} = 5.02$ TeV in [25], where no significant deviations are found

The ratio of jet production cross sections reconstructed with $R = 0.2$ and 0.4 is shown in Fig. 6. For all centrality classes, the ratio shows the expected stronger jet collimation towards higher $p_\mathrm {T}$. Moreover, the ratio is for all centralities consistent with the result obtained in minimum bias  p–Pb   collisions, which agrees with the jet cross section ratio in $\mathrm{pp}$ collisions as shown in [25]. The result is fully compatible with the expectation, since even in central  Pb–Pb collisions, where a significant jet suppression in the nuclear modification factor is measured, the cross section ratio remains unaffected [15].

## Summary

Centrality-dependent results on charged jet production in  p–Pb  collisions at $\sqrt{s_\mathrm {NN}} = 5.02$ TeV have been shown for transverse momentum range $20 < p_{\mathrm {T,\,ch\;jet}} < 120~\text{ GeV/ }c$ and for resolution parameters $R=0.2$ and $R = 0.4$. The centrality selection is performed using the forward neutron energy, and the corresponding number of binary collisions $N_\mathrm {coll}$ is estimated via the correlation to the multiplicity measured in the lead-going direction, in order use a rapidity region well separated from the one where jets are reconstructed.

With this choice of centrality and data driven $N_\mathrm {coll}$ estimate, the nuclear modification factor $Q_\mathrm {pPb}$ is consistent with unity and does not indicate a significant centrality dependence within the statistical and systematical uncertainties. In the measured kinematic range momentum between 20 $\text{ GeV/ }c$ and up to 120 $\text{ GeV/ }c$ and close to mid-rapidity, the observed nuclear modification factor is consistent with results from full jet measurements by the ATLAS collaboration in the same kinematic region. The jet cross section ratio for $R=0.2$ and 0.4 shows no centrality dependence, indicating no modification of the degree of collimation of the jets at different centralities.

These measurements show the absence of strong nuclear effects on the jet production at mid-rapidity for all centralities.
